# Supplementary material for: Living with vaccine-induced immune thrombocytopenia and thrombosis: a qualitative study
Source: BMJ Open. 2023 Jul 9;13(7):e072658. doi: 10.1136/bmjopen-2023-072658 (PMC10335444; doi:10.1136/bmjopen-2023-072658)
Supplement: Supplementary data [file bmjopen-2023-072658supp001.pdf]

Topic guide

Record!

Check consent - aware that is being recorded, can drop out at any time, audio recordings will be destroyed and anonymized data used in research outputs.

Age

Gender

Job

Single/married/partner

Want to discuss the impact of VITT on you

1. Briefly, what experiences during initial onset of symptoms/hospitalization?

2. What were the psychological consequences of these experiences at the time for you?

   Prompt

   - Impact of isolation due to covid
   - Fear, worry

3. What physical symptoms had since then? How have these impacted on your life?

   Prompt

   - Day to day – mobility, pain, fatigue restrictions
   - Longer term – finance, job
   - Impact on family

4. What were the longer-term psychological consequences of living with VITT?

   Prompt

   - Health anxiety
   - Depression
   - Anger/frustration

5. *If time*, what are views on vaccination/covid now?

Thanks for participating. Reminder that if need support get in touch with Thrombosis UK on email in the PIS.

Will send copy of findings
